# Mechanistic Insight into the Host Transcription Inhibition Function of Rift Valley Fever Virus NSs and Its Importance in Virulence

**DOI:** 10.1371/journal.pntd.0005047

**Published:** 2016-10-06

**Authors:** Kaori Terasaki, Sydney I. Ramirez, Shinji Makino

**Affiliations:** 1 Department of Microbiology and Immunology, The University of Texas Medical Branch, Galveston, Texas, United States of America; 2 Department of Pathology, The University of Texas Medical Branch, Galveston, Texas, United States of America; 3 Center for Biodefense and Emerging Infectious Diseases, The University of Texas Medical Branch, Galveston, Texas, United States of America; 4 UTMB Center for Tropical Diseases, The University of Texas Medical Branch, Galveston, Texas, United States of America; 5 Sealy Center for Vaccine Development, The University of Texas Medical Branch, Galveston, Texas, United States of America; 6 The Institute for Human Infections and Immunity, The University of Texas Medical Branch, Galveston, Texas, United States of America; Naval Medical Research Center, UNITED STATES

## Abstract

Rift Valley fever virus (RVFV), a member of the genus *Phlebovirus* within the family *Bunyaviridae*, causes periodic outbreaks in livestocks and humans in countries of the African continent and Middle East. RVFV NSs protein, a nonstructural protein, is a major virulence factor that exhibits several important biological properties. These include suppression of general transcription, inhibition of IFN-β promoter induction and degradation of double-stranded RNA-dependent protein kinase R. Although each of these biological functions of NSs are considered important for countering the antiviral response in the host, the individual contributions of these functions towards RVFV virulence remains unclear. To examine this, we generated two RVFV MP-12 strain-derived mutant viruses. Each carried mutations in NSs that specifically targeted its general transcription inhibition function without affecting its ability to degrade PKR and inhibit IFN-β promoter induction, through its interaction with Sin3-associated protein 30, a part of the repressor complex at the IFN-β promoter. Using these mutant viruses, we have dissected the transcription inhibition function of NSs and examined its importance in RVFV virulence. Both NSs mutant viruses exhibited a differentially impaired ability to inhibit host transcription when compared with MP-12. It has been reported that NSs suppresses general transcription by interfering with the formation of the transcription factor IIH complex, through the degradation of the p62 subunit and sequestration of the p44 subunit. Our study results lead us to suggest that the ability of NSs to induce p62 degradation is the major contributor to its general transcription inhibition property, whereas its interaction with p44 may not play a significant role in this function. Importantly, RVFV MP-12-NSs mutant viruses with an impaired general transcription inhibition function showed a reduced cytotoxicity in cell culture and attenuated virulence in young mice, compared with its parental virus MP-12, highlighting the contribution of NSs-mediated general transcription inhibition towards RVFV virulence.

## Author Summary

Rift Valley fever virus (RVFV) has a significant impact on the livestock industry because of its high mortality rate in young ruminants and causation of a high abortion rate in pregnant animals. Human RVFV infections generally manifest as self-limiting and non-fatal illnesses. However, a small percentage of patients develop encephalitis, vision loss and hemorrhagic fever with a high mortality rate. Currently, there is no commercially available vaccine for human use or effective antiviral drug for RVFV treatment. The non-structural protein NSs is a major virulence factor of RVFV, which mediates suppression of host general transcription, inhibition of IFN-β transcription and degradation of PKR, to block host antiviral responses. To examine the contribution of host transcription inhibition to RVFV virulence, we generated RVFV MP-12 strain-derived mutants that have attenuated inhibitory activity on host transcription due to amino acid mutations in NSs. The mutant viruses showed attenuated cytotoxicity in cell culture and attenuated virulence in young mice, demonstrating the contribution of NSs-mediated host transcription inhibition to the virulence of RVFV.

## Introduction

Rift Valley fever virus (RVFV) is the pathogen causing Rift Valley fever, which affects both humans and domestic ruminants, primarily in countries of the African continent and Middle East. The virus is an arbovirus and circulates between mosquito vectors and ruminants in endemic areas. RVFV causes high mortality rates in young ruminants and a high rate of abortions in pregnant ruminants [1]. Humans are infected with the virus either by mosquito bite or by direct contact with materials of infected animals. The majority of patients show influenza-like symptoms but few develop hemorrhagic fever, neurological symptoms, and ocular disease [2]. Due to its major impact on public health, RVFV is classified as a category A priority pathogen by the National Institute of Allergy and Infectious Diseases. Currently there is no approved vaccine available for humans and animals in non-endemic areas.

RVFV belongs to the family *Bunyaviridae*, genus *Phlebovirus*. RVFV is an enveloped virus and carries 3 segmented RNA genomes, the L, M and S segments, which are of negative or ambisense polarity. The L segment encodes L protein, a viral RNA-dependent RNA polymerase. M RNA encodes 78kDa protein, NSm protein, Gn protein and Gc protein, the latter two of which are major envelope glycoproteins and generated by co-translational cleavage of precursor Gn/Gc polyprotein. 78kDa protein is dispensable for virus replication [3], whereas it plays important roles in virus dissemination in mosquitoes [4, 5]. NSm is a viral anti-apoptotic protein [6, 7] and also is important for efficient virus replication in macrophage cell lines [5]. S RNA expresses a nucleocapsid (N) protein and a nonstructural protein NSs by using an ambisense coding strategy. The N protein encapsidates the viral RNA and forms a ribonucleocapsid complex with L protein [8].

RVFV NSs protein is a phosphoprotein with an apparent molecular weight of 31 kDa and is localized in both the cytoplasm and nucleus [9]. In the nucleus, NSs forms filament-like structures by self-dimerization through its C-terminal domain [10]. RVFV NSs protein is a major virus virulence factor and has various important biological functions, which are important for countering the host antiviral response. One of the NSs functions is suppression of IFN-β mRNA transcription. NSs binds to Sin3-associated protein 30 (SAP30), a subunit of a co-repressor complex, and maintains IFN-β promoter in a transcriptionally silent state, leading to suppression of IFN-β mRNA transcription [11]. In addition to the specific inhibition of IFN-β transcription, NSs suppresses general transcription; it has been proposed that NSs exerts suppression of general transcription by interacting with subunits of transcription factor II H (TFIIH) complex, p44 and p62 [12, 13]. A recent study showed that the NSs-mediated general transcription inhibition contributes to the inhibition of IFN-β mRNA transcription [14]. Although NSs-mediated transcription suppression has been considered to be important in suppressing the host antiviral response, the exact effects of the NSs-mediated general transcription inhibition on virus virulence have not been defined. NSs promotes the degradation of double-stranded RNA-dependent protein kinase R (PKR), an antiviral IFN-stimulated gene product, through a proteasome pathway to prevent phosphorylation of eIF2-α triggered by RVFV infection [15–18]. Furthermore, NSs contributes to cellular stress responses such as an increase in reactive oxygen species, activation of DNA damage signaling, cell cycle arrest, and activation of the p53 signaling pathway [19–22]. Although how NSs induces the cellular stresses remains largely unknown, these stress responses may contribute to RVFV-induced cell death.

To elucidate the mechanisms of the different functions of NSs, it would be of great value to characterize a series of NSs mutants, each of which specifically lacks one of these NSs functions but retains other functions. However, introducing any short in-frame deletion in the RVFV NSs resulted in loss of all functions [23], suggesting to us that the NSs protein structure is important for its biological activity. Hence, it has been challenging to generate NSs mutants that lack a specific biological function but retain its other functions. The lack of these NSs mutants has prevented a detailed mechanistic analysis of each biological function of the NSs. In this study, we generated two RVFV mutants, each carrying mutations in NSs, that specifically targeted its general transcription inhibition function, to delineate the mechanism of its inhibition by NSs. Furthermore, we examined the importance of the NSs-mediated inhibition of general transcription on virus-induced cytotoxicity and tested its role in RVFV virulence by using a young mouse model.

## Materials and Methods

### Ethics statement

All mouse studies were performed in facilities accredited by the Association for Assessment and Accreditation of Laboratory Animal Care in accordance with the recommendations in the Guide for the Care and Use of Laboratory Animals (Institute of Laboratory Animal Resources, National Research Council, National Academy of Sciences, 1996). The animal protocol (protocol number, 1105023A) was approved by the Institutional Animal Care and Use Committee of The University of Texas Medical Branch.

### Plasmid constructions

A standard recombinant PCR method, in which pProT7-S encoding antiviral-sense S RNA [24] served as a template, was used to generate pProT7-S carrying mutations in NSs coding region. The NSs coding region of pProT7-S was amplified by using primers carrying Mfe I or a Not I site. After digestion with Mfe I and Not I, the PCR fragment was cloned into Eco RI and the Not I site of a pCAGGS plasmid, resulting in the plasmids expressing NSs protein. For expression of human FBXO3 isoform 1 (FBXO3/1) [14], intracellular RNAs of MRC-5 cells were subjected to cDNA synthesis, and the FBXO3/1 gene was amplified with primers carrying Mfe I or the Not I site. After addition of an N-terminal V5 tag, the PCR product was cloned into EcoR I and the Not I site of pCAGGS. For expression of human SAP30, PCR product encoding human SAP30 with N-terminal V5 tag was cloned into EcoR I and the Xho I site of pCAGGS. All of the constructs were confirmed by sequencing.

### Cells and viruses

BSR-T7/5 cells which stably express T7 RNA polymerase [25] were maintained in Glasgow’s minimal essential medium (MEM) (Lonza) containing 10% fetal bovine serum (FBS), 10% tryptose phosphate broth, MEM Amino Acids Solution and geneticin (1 mg/ml). Vero E6 cells were maintained in Dulbecco’s modified MEM (Gibco) containing 5% FBS. MRC-5 cells were maintained in Eagle's MEM (EMEM) (Gibco) containing 10% FBS, MEM Non-Essential Amino Acids Solution (Gibco), and 1% sodium pyruvate (Sigma). HeLa cells were maintained in EMEM containing 10% FBS. MP-12 is a highly attenuated RVFV strain obtained after 12 serial passages of an RVFV ZH548 strain in the presence of 5-fluorouracil [26]. A recombinant MP-12 strain and other MP-12-derived mutants were rescued from cDNAs as described previously [24], except that BSR-T7/5 cells were used in place of BHK/T7-9 cells. Titers of the rescued viruses were determined by using a plaque assay [24]. For the virus carrying R16H/M250K mutations in NSs, passage 0 (P0) virus obtained from plasmid-transfected BSR-T7/5 cells were serially diluted and inoculated into VeroE6 cells. The highest titer of P1 virus was selected and used for the study.

### Antibodies

Anti-PKR rabbit polyclonal antibody, anti-Flag tag mouse monoclonal antibody, anti-Flag tag rabbit monoclonal antibody, and anti-V5 tag rabbit monoclonal antibody were purchased from Cell Signaling Technology. Anti-GTF2H1 (p62) mouse monoclonal antibody, anti-GTF2H2 (p44) mouse polyclonal antibody, and anti-XPD mouse monoclonal antibody were purchased from Abcam. Anti-TFIIH p44 (N-17) goat polyclonal antibody, anti-β-actin goat polyclonal antibody and horseradish peroxidase (HRP)-labeled anti-mouse, anti-goat, and anti-rabbit secondary antibodies were purchased from Santa Cruz Biotechnology. Anti-GST-N rabbit polyclonal antibody was generated by inoculating a rabbit with GST-N fusion protein (the entire N protein was fused with the C terminus of GST protein) followed by affinity purification of the serum [3]. Anti-MP-12 mouse serum was provided by Dr. Robert B. Tesh at The University of Texas Medical Branch. The monoclonal antibody H2K^k^D^k^ (H2K), which is against major histocompatibility complex class I antigen, was obtained from Dr. Paul Gottlieb at The University of Texas at Austin.

### Western blot analysis

Cells were washed with phosphate-buffered saline (PBS) and suspended in SDS polyacrylamide gel electrophoresis (SDS-PAGE) sample buffer. Samples were boiled for 3–5 min and subjected to SDS-PAGE. Proteins were electroblotted onto polyvinylidene difluoride membranes (immune blot: Bio Rad). After blocking with skim milk, the membranes were incubated with the primary antibody for 1 h at room temperature and with the secondary antibody for 1 h at room temperature. The proteins on the membrane were detected by using an ECL Western Blotting Detection Reagent (GE Healthcare Life Sciences) or ECL plus Western Blotting Substrate (Pierce).

### Metabolic radiolabeling of intracellular proteins

Vero E6 cells were infected with either MP-12 or its mutants at a multiplicity of infection (m.o.i.) of 3. At 16 h post infection (p.i.), the culture media was replaced with methionine/cysteine-free medium. After starvation for 30 min, the infected cells were labeled with 100 μCi/ml of ^35^S-methionine/cysteine (1,000 Ci/mmol; MP Biomedicals) for 1 h. The radiolabeled cells were suspended in 2x SDS-PAGE sample buffer, resolved by SDS-PAGE and visualized by Coomassie Blue staining or autoradiography.

### Detection of global RNA transcription

The Click-iT RNA Alexa Fluor 488 Imaging Kit was purchased from Thermo Fisher Scientific. Vero E6 cells were infected with recombinant viruses at an m.o.i. of 3 and incubated with 1 mM of 5-ethynyl uridine (5EU) for 1 h at 16 h p.i. Cellular RNA was stained with Alexa fluor, 488-coupled azide for 30 min by following the manufacturer's protocol. For a negative control, mock-infected cells were treated with 5 μg/ml of actinomycin D (ActD) for 30 min prior to 5EU treatment and for 1 h during 5EU treatment. After the click reaction, RVFV N protein was stained with anti-GST-N rabbit polyclonal antibody followed by Alexa fluor 594 conjugated anti-rabbit antibody. Images were captured on a Zeiss Axiophot 2 fluorescence microscope with a 40x magnification lens and processed with the ImageJ software [27]. For flow cytometry analysis, the infected cells were detached from the dish by Accumax (Innovative Cell Technologies) after the 5EU treatment and suspended in culture media. After washing with PBS containing 1% bovine serum albumin (BSA), cells were fixed with 2% formaldehyde/PBS for 30 min at room temperature and blocked with blocking buffer (PBS containing 0.2% saponin and 1% BSA) for 15 min on ice. Cellular RNA was stained by Alexa fluor 488-coupled azide for 30 min in the presence of 0.5% saponin and 1% BSA. After washing with the blocking buffer, RVFV N protein was stained by anti-GST-N rabbit polyclonal antibody for 30 min on ice followed by Alexa fluor 594 conjugated anti-rabbit antibody. The cells were washed with PBS containing 1% BSA, passed through a cell strainer (BD Falcon), and analyzed on an LSRII Fortessa (BD Biosciences). Single cells were gated based on their forward scatter and side scatter profile. More than 30,000 count of the gated single cells were analyzed for each experiment.

### Cell viability assay

Confluent Vero E6 cells grown in 96-well plates were either mock infected or with mutant virus at an m.o.i. of 3. Cell viability was determined by using Viral ToxGlo (Promega), which measures cellular ATP. At various times p.i., ATP detection reagent was added to each well. After incubation for 10 min, luminescence was measured by SpectraMax M5e (Molecular Devices).

### Northern blot analysis

Total RNAs were extracted by using TRIzol reagent (Invitrogen) and subjected to Northern blot analysis as described previously [28]. Strand-specific digoxigenin-labeled RNA probes and a digoxigenin (DIG) system (Roche) were used to detect RNA. The 564-nucleotide-long, 293 cell-derived, and DIG-labeled IFN-β riboprobe [29] was used for IFN-β mRNA detection.

### Indirect immunofluorescence assay

Cells cultured on chamber slides were fixed in 4% paraformaldehyde for 15 min. The fixed cells were permeabilized with 0.2% TritonX-100 for 15 min and blocked with 1% BSA in PBS for 30 min. After the blocking, the cells were stained with primary antibody diluted with the blocking solution, followed by incubation with Alexa Fluor 488 or 594-conjugated secondary antibodies (Molecular Probes). Images were captured by a Zeiss Axiophot 2 fluorescence microscopy and processed with ImageJ software [27].

### Co-immunoprecipitation analysis

To detect the interaction between NSs and p44, we infected HeLa cells with MP-12-NSs-Flag, which expresses NSs with the C-terminal Flag tag or its mutant viruses at an m.o.i. of 3. At 8 h p.i., the cells were lysed in lysis buffer [50 mM Tris-HCl, pH 7.6, 0.1% NP-40, 150 mM NaCl, 1 mM EDTA, protease inhibitors (Sigma), and 100 U/ml Benzonase (Sigma)]. After 3 cycles of freeze-thaw, the cell lysate was cleared by centrifugation at 4°C and 100,000 x g for 1 h and incubated with Dynabeads protein G (Life Technologies) conjugated with anti-GTF2H2 (p44) mouse monoclonal antibody or H2K antibody according to the manufacturer’s protocol. Precipitates were washed with the lysis buffer with 0.1% Tween 20 and analyzed by Western blotting using the anti-p44 goat polyclonal, and anti-p44 mouse monoclonal and anti-Flag mouse monoclonal antibodies. To detect the interaction between NSs and SAP30, we transfected HeLa cells with pCAGGS-V5-SAP30 which encodes human SAP30 carrying an N-terminus V5 tag by using FuGENE HD transfection reagent (Promega). At 16 h post transfection, the cells were infected with MP-12-NSs-Flag or its mutant viruses. The cells were lysed with lysis buffer (50 mM Tris-HCl, pH 7.5, 100 mM NaCl, 1% NP-40, 1 mM EDTA, and protease inhibitor cocktail) [11] at 8 h p.i. The cell lysate was cleared by centrifuge at 21,000 x g and 4°C for 15 min and incubated with Anti-V5-tag mAb-Magnetic Beads (MBL International) according to the manufacturer’s protocol. Precipitates were washed with the lysis buffer containing 450 mM NaCl and analyzed by Western blotting by using anti-V5 tag rabbit monoclonal and anti-Flag mouse monoclonal antibodies. For detection of interaction between NSs and p62, HeLa cells were infected with MP-12-NSs-Flag or its mutant viruses at an m.o.i. of 3. At 5 h p.i., the cells were lysed in lysis buffer [20 mM Tris-HCl, pH 7.6, 0.1% TritonX-100, 150 mM NaCl, 1 mM EDTA, protease inhibitors (Sigma), and 100 U/ml Benzonase (Sigma)] [13]. After 3 cycles of freeze-thaw, the cell lysate was cleared by centrifugation at 4°C and 100,000 x g for 1 h and incubated with Dynabeads protein G conjugated with anti-GTF2H1 (p62) mouse monoclonal antibody according to the manufacturer’s protocol. Precipitates were analyzed by Western blotting using the anti-p62 mouse monoclonal and anti-Flag mouse monoclonal antibodies. To detect the interaction between NSs and FBXO, we transfected HeLa cells with pCAGGS-V5-FBXO3/1, which encodes FBXO3/1 carrying an N-terminus V5 tag, by using FuGENE HD transfection reagent. At 16 h post transfection, the cells were infected with MP-12-NSs-Flag or its mutant viruses and lysed with lysis buffer (50 mM Tris-HCl, pH 7.5, 0.2% NP-40, 5% glycerol, 100 mM NaCl, 1.5 mM MgCl_2_, and protease inhibitor cocktail) [14] at 5 h p.i. The cell lysate was cleared by centrifugation at 21,000 x g and 4°C for 15 min and incubated with Anti-V5-tag mAb-Magnetic Beads (MBL International) according to the manufacturer’s protocol. Precipitates were washed with the lysis buffer and analyzed by Western blotting by using the anti-V5 tag rabbit monoclonal and anti-Flag mouse monoclonal antibodies.

### Renilla luciferase reporter assay

HeLa cells prepared in 12-well plates were transfected with 0.25 μg of the pCAGGS-based plasmid encoding either NSs or mutant NSs or an empty vector along with 0.1 μg of pRL-SV40 (Promega) which expresses Renilla luciferase under control of an SV40 promotor. Renilla luciferase activities in the transfected cells were measured by using a Renilla luciferase assay system (Promega) at 24h post transfection.

### Testing virulence of MP-12-derived mutant viruses in young mice

Eighteen-day-old CD-1 mice were intraperitoneally inoculated with 10^4^ PFU of MP-12, MP-12-M250K or MP-12-R16H/M250K or with Hank's Balanced Salt Solution (HBSS). The animals were observed for survival for 22 days post inoculation.

## Results

### Isolation of novel RVFV NSs mutants

We previously generated and characterized an RVFV MP-12 strain-derived mutant virus, which is deficient for efficient virus genome co-packaging due to a large deletion in the 5’ untranslated region of M RNA segment [30]. To obtain virus variants, carrying mutations that can compensate for this deficiency, we serially passaged this mutant virus in type I IFN-incompetent Vero E6 cells; the virus inoculum, used for each passage, was diluted 10 times and the released viruses were harvested at 4 days p.i. The titer of the mutant virus progressively increased with each passage; at passage 18, the virus titer was 2.9 x 10^6^ PFU/ml, which was 2 logs higher than the initial titer of 2.0 x 10^4^ PFU/ml prior to the serial passage. We isolated 34 plaque-cloned viruses from passage level 18, and 28 had a large deletion in the NSs gene in the S segment, whereas 6 plaque-cloned viruses retained the full-length NSs gene. We determined the full genome sequence of 5 clones that retained the full-length NSs gene and found that all of the isolated viruses had several mutations in the L, M, and S segments. Table 1 shows mutations found in the NSs genes of 5 plaque-cloned viruses and the uncloned passage 18 virus. All five plaque-cloned viruses and the passage 18 virus had an amino-acid substitution at position 250, including M250K or M250T mutations. Two plaque-cloned viruses and the passage 18 virus had a D100G substitution. Other mutations found in plaque-cloned viruses were R16H and A75E. Although K202N mutation was found in the uncloned passage 18 virus sample, this mutation was absent in the plaque-cloned viruses.

**Table 1:** Nucleotide and amino acid mutations in the NSs gene of uncloned and plaque-cloned passage 18 virus.

	Nucleotide*	Amino acid
**Clone1**	**A908U**	**M250K**
	**G1174A**	
**Clone2**	**A908U**	**M250K**
**Clone3**	**A908G**	**M250T**
	**U1358C**	**D100G**
**Clone4**	**A908G**	**M250T**
	**U1358C**	**D100G**
	**G1433U**	**A75E**
	**C1504U**	
**Clone5**	**A908U**	**M250K**
	**C1610U**	**R16H**
**Uncloned**	**A908U**	**M250K**
	**A908G**	**M205T**
	**U1051A**	**K202N**
	**U1358C**	**D100G**

*Number denotes the position in anti-genomic strand

**Fig 1:**
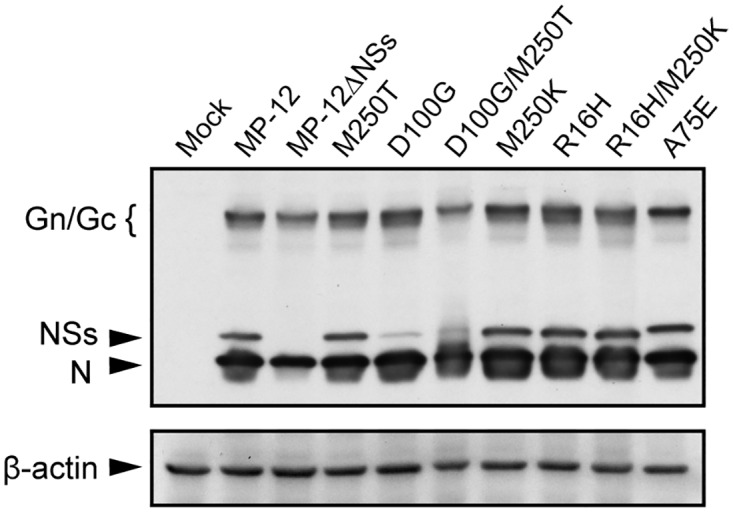
Accumulation of wt and mutant NSs proteins in infected cells. Vero E6 cells were mock infected (Mock) or infected with MP-12 or MP-12 mutants, each carrying a different NSs mutant, at an m.o.i. of 3. The infected cells were harvested at 16 h p.i. and analyzed in Western blots by using anti-MP-12 antibody or anti-β actin antibody followed by secondary antibody.

To test whether the mutations affect NSs functions, we generated MP-12-based mutant viruses, each carrying R16H, R16H+M250K (NSs-R16H/M250K), A75E, D100G, D100G+M250T, M250K (NSs-M250K), or M250T mutations in the NSs, by using a reverse genetics system [24]. Accumulations of NSs protein in VeroE6 cells infected with MP-12 carrying the D100G mutation or the D100G+M250T mutations were significantly lower than those in MP-12 infected cells (Fig 1), indicating that the D100G mutation affected efficient NSs accumulation. Due to the poor accumulation of NSs, we excluded the MP-12 mutant carrying the D100G mutation and that carrying the D100G+M250T mutation from subsequent analysis. In cells infected with the other mutants, levels of NSs protein accumulation were similar to that in MP-12 infected cells. In this study, we refer to MP-12 NSs as wild type NSs (wt NSs).

### Effect of mutations in NSs on general host transcription

**Fig 2:**
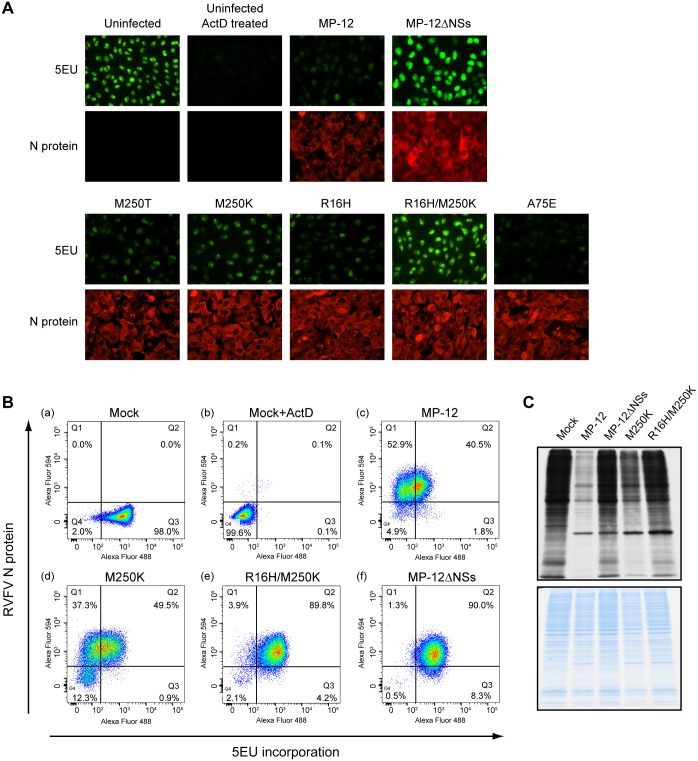
Effects of NSs mutants on host general transcription. (A) Fluorescence microscopic analysis of newly synthesized RNAs. Vero E6 cells were mock infected (uninfected), mock infected and ActD treated (Uninfected ActD treated) or infected with MP-12 or its mutants at an m.o.i. of 3. At 16 h p.i., cells were incubated in the presence of 5EU for 1 h. The incorporated 5EU were visualized by using Alexa fluor 488 conjugated azide (5EU), and viral N protein was stained by anti-N antibody followed by Alexa fluor 594 conjugated secondary antibody (N protein). Fluorescence microscopic analysis was performed to detect newly synthesized RNAs. (B) Flow cytometric analysis of newly synthesized RNAs in infected cells. Vero E6 cells were mock infected, mock infected and ActD treated, or infected with MP-12, MP-12-M250K, or MP-12-R16H/M250K, at an m.o.i. of 3. 5EU and anti-N antibody were used to label newly synthesized RNAs and N proteins, respectively, as described in (A). Then the cells were subjected to Flow cytometric analysis. The density dot plot was divided into four quadrants (Q1, Q2, Q3, and Q4). Quadrant Q1 (Upper left quadrant): RVFV N protein positive and low activity of RNA transcription. Quadrant Q2 (Upper right quadrant): RVFV N protein positive and high activity of RNA transcription. Quadrant Q3 (Lower right quadrant): RVFV N protein negative and high activity of RNA transcription. Quadrant Q4 (Lower left quadrant): RVFV N protein negative and low activity of RNA transcription. (C) Autoradiography of radiolabeled proteins in mock infected cells and infected cells. Vero E6 cells were mock infected (mock) or infected with MP-12 or its mutants at an m.o.i. of 3. At 16 h p.i., cells were incubated with ^35^S-methionine/cysteine for 1 h. Whole cell lysates were resolved by SDS-PAGE and visualized by autoradiography (top panel) or Coomassie Blue staining (bottom panel).

To evaluate host transcriptional shut-off activities of these NSs mutants, levels of RNA synthesis in virus-infected VeroE6 cells were measured by labeling with 5EU from 16 h to 17 h p.i. Mock-infected cells alone, as well as those treated with ActD, and MP-12-infected cells and cells infected with MP-12ΔNSs, which lacks the NSs gene [24], served as controls. 5EU-labeled RNA, which was detected as a fluorescence signal, was mainly observed in the nucleus but not in the cytoplasm, indicating that viral RNA synthesis, which occurs exclusively in the cytoplasm, was not very active at 16h post infection. As shown in Fig 2A, strong signals of fluorescent-labeled RNA were observed in the nucleus of mock-infected cells and MP-12ΔNSs-infected cells, demonstrating active host RNA synthesis. In contrast, ActD-treated cells showed very weak fluorescent signals, demonstrating ActD-mediated host transcriptional shut-off. MP-12-infected cells showed weaker fluorescent signals in the nucleus, compared with those in MP-12ΔNSs-infected cells, demonstrating an inhibition of host transcription by NSs. Fluorescent intensity observed in cells infected with virus carrying mutation M250T, R16H or A75E in NSs was similar to that in MP-12-infected cells, suggesting that host transcription was still inhibited by these NSs mutants. In contrast, cells infected with MP-12 carrying NSs-R16H/M250K (MP-12-R16H/M250K) had bright fluorescent signals, whose intensities were similar to those in mock-infected cells or MP-12ΔNSs-infected cells. Fluorescent signal intensities of cells infected with MP-12 carrying NSs-M250K (MP-12-M250K) were between those of MP-12-infected cells and MP-12ΔNSs-infected cells.

We next used flow cytometry analysis to quantitatively measure the levels of 5EU-labeled RNAs in MP-12-M250K-infected cells and in MP-12-R16H/M250K-infected cells. We used the same controls as described above. Fig 2B shows the results using density plots, wherein 5EU incorporation and N protein levels are shown on the X and Y axes, respectively. In mock-infected and ActD-treated samples, most of the cells were found in quadrant 3 (Q3) and Q4, respectively, demonstrating active host transcription in the former, but not in the latter (Fig 2B-a and 2B-b). In all infected samples, 86.8–93.4% of cells were RVFV N protein positive (Q1+Q2), demonstrating that most of the cells were infected. The level of 5EU-labeled RNA in MP-12ΔNSs-infected cells was similar to that in mock-infected cells, demonstrating similar RNA synthesis activity in these two samples (Fig 2B-a and 2B-f). MP-12-infected cells showed lower levels of 5EU-labeled RNA compared with those in MP-12ΔNSs infected cells, demonstrating its lower RNA transcription activity (Fig 2B-c and 2B-f). MP-12-R16H/M250K-infected and MP-12ΔNSs-infected cells showed similar density plot patterns, suggesting to us that host transcription inhibition did not occur in MP-12-R16H/M250K-infected cells (Fig 2B-e and 2B-f). MP-12-M250K-infected cells showed lower levels of 5EU-labeled RNA compared with those in MP-12ΔNSs-infected cells (Fig 2B-d and 2B-f). However, the percentage of MP-12-M250K-infected cells with low transcription activity was 43.0% (Q1/Q1+Q2), while in the MP-12 samples, it was 56.6%. These results suggested that MP-12-M250K replication suppressed host general transcription, and yet the inhibitory activity of MP-12-M250K was weaker than that of MP-12.

We next examined the effect of differences in the transcription inhibitory activities of the mutant viruses on global protein synthesis by incorporation of ^35^S-methionine/cysteine into newly synthesizing proteins in mock-infected cells and in cells infected with MP-12, MP-12ΔNSs, MP-12-M250K, or MP-12-R16H/M250K (Fig 2C). Consistent with our previous study [24], MP-12 replication, but not MP-12ΔNSs replication, suppressed host protein synthesis. MP-12-R16H/M250K replication did not inhibit host protein synthesis, while MP-12-M250K replication moderately inhibited host protein synthesis.

Taken together, these analyses showed that NSs-R16H/M250K lost the general transcription suppression activity, leading to efficient host protein synthesis in infected cells, while NSs-M250K exerted inefficient general transcription suppression activity, causing moderate levels of host protein synthesis inhibition in infected cells.

### Mutations in NSs attenuated MP-12-induced cytotoxicity

**Fig 3:**
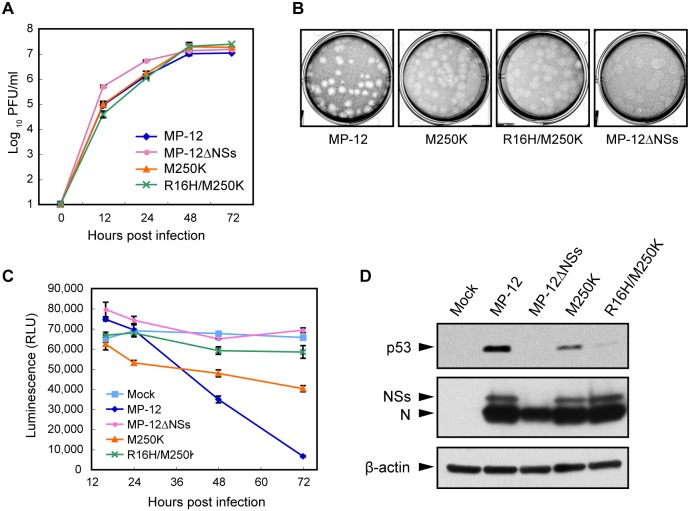
Attenuated cytotoxicity of MP-12 NSs mutants. (A) Growth kinetics of MP-12 and its mutants. Vero E6 cells were infected with each virus at an m.o.i. of 0.01, and the culture supernatants were collected at various times p.i. Virus titers were determined by plaque assays. (B) Plaque morphology of the MP-12 and mutant viruses in VeroE6 cells at 3 day p.i. (C) Viability of cells infected with MP-12 or its mutants infected cells. Vero E6 cells were mock infected or infected with each virus at an m.o.i. of 3. Cell viability was determined by measuring cellular ATP. (D) Abundance of p53 in infected cells. Vero E6 cells were infected with each virus at an m.o.i. of 3 and harvested at 24 h p.i. Whole cell lysate was subjected to Western blot analysis. Anti-p53 antibody, anti-MP-12 antibody and anti-β-actin antibody were used as the primary antibody for the top, middle and bottom panels, respectively.

Analysis of replication kinetics of MP-12, MP-12ΔNSs, MP-12-R16H/M250K and MP-12-M250K showed that all viruses replicated efficiently with similar replication kinetics in Vero E6 cells (Fig 3A).

MP-12 formed clear plaques in Vero E6 cells, whereas MP-12-R16H/M250K and MP-12-M250K formed turbid-type plaques like MP-12ΔNSs (Fig 3B), indicating that the R16H/M250K mutation and the M250K mutation in NSs affected virus-induced cytotoxicity. Cell viability assays showed that MP-12 replication caused a 90% reduction in cell viability as compared with mock-infected cells at 72 h p.i. (Fig 3C), whereas MP-12ΔNSs-infected cells and mock-infected cells showed similar cell viabilities throughout the course of the experiments. MP-12-R16H/M250K-infected cells and MP-12-M250K-infected cells showed an 11% and 39% decrease in cell viability, respectively, as compared with that in mock-infected cells at 72 h p.i., demonstrating that both of the mutant viruses were less cytotoxic than MP-12. MP-12, MP-12-M250K and MP-12-R16H/M250K had similar replication kinetics, regardless of their differential ability to induce cytotoxicity (Fig 3), implying that virus-induced cytotoxicity did not have a significant impact on RVFV replication kinetics in Vero E6 cells.

Because others reported that MP-12 replication induces NSs-dependent p53 stabilization, which contributes to virus-induced cell death [21], we next examined stabilization of p53 in cells infected with MP-12 and other mutant viruses (Fig 3D). Amounts of p53 protein significantly increased in MP-12-infected cells, but not in MP-12ΔNSs-infected cells, confirming the NSs-dependent p53 stabilization in the infected cells. Accumulation of p53 also occurred in MP-12-M250K-infected cells, whereas the accumulation levels were lower than those in MP-12-infected cells. Very low levels of p53 accumulation occurred in MP-12-R16H/M250K-infected cells.

### Effect of R16H/M250K and M250K mutations in NSs on its PKR degradation and IFN-β inhibition activities

**Fig 4:**
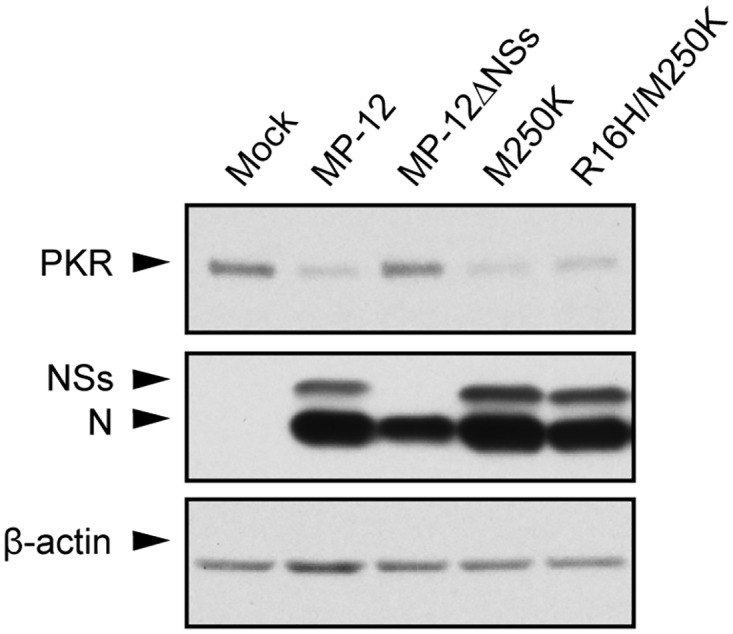
Effects of M250K and R16H/M250K mutations on PKR stability in infected cells. HeLa cells were infected with MP-12 or mutant viruses at an m.o.i. of 3. Whole-cell lysate was harvested at 8 h p.i. and subjected to Western blot analysis by using anti-PKR antibody, anti-MP-12 antibody and anti-β-actin antibody as the primary antibody for the top, middle and bottom panels, respectively.

NSs interacts with PKR and triggers degradation of the PKR by a proteasome pathway [15–18]. To investigate whether the NSs mutants retain the ability for PKR degradation, we compared the total amount of PKR in virus-infected cells. Similar levels of reduction in the amounts of PKR occurred in cells infected with MP-12, MP-12-M250K or MP-12-R16H/250K, suggesting that NSs-R16H/M250K and NSs-M250K promoted PKR degradation as efficiently as wt NSs (Fig 4).

**Fig 5:**
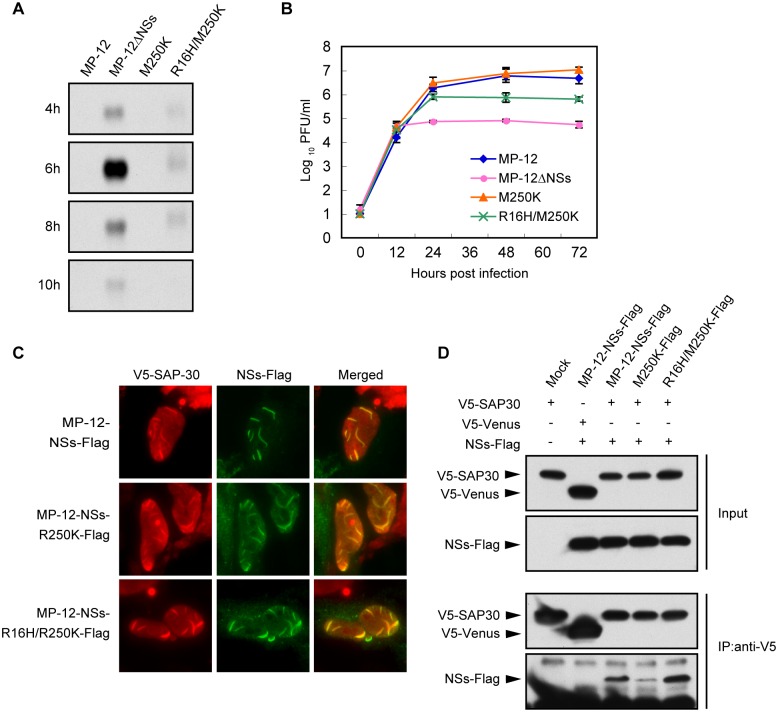
Inhibition of IFN-β mRNA expression by MP-12 NSs mutants. (A) MRC-5 cells were infected with MP-12 or its mutants at an m.o.i of 3. Total RNA was collected at various time p.i. as indicated. IFN-β mRNA was detected by Northern blotting analysis using the IFN-β mRNA-specific RNA probe. (B) Growth kinetics of MP-12 and its mutants in MRC-5 cells. MRC-5 cells were infected with each virus at an m.o.i. of 0.01, and the culture supernatants were collected at various times p.i. Virus titers were determined by using a plaque assay in Vero E6 cells. (C) Co-immunostaining for NSs and SAP30. HeLa cells were transfected with plasmid expressing human SAP30 carrying an N-terminal V5 tag and infected with MP-12 carrying a Flag-tagged NSs or mutant MP-12 carrying Flag-tagged NSs mutant at 16 h post transfection. At 6 h p.i., the cells were fixed, immunostained with an anti-V5 tag and anti-Flag tag antibodies, and subjected to fluorescent microscopic examination. (D) Co-immunoprecipitation of NSs and SAP30. HeLa cells were transfected with the plasmid encoded V5-tagged SAP30 or V5-tagged Venus protein. At 16 h post transfection, cells were mock infected or infected with the MP-12 carrying Flag-tagged NSs or mutant MP-12 carrying Flag-tagged NSs mutant. At 8 h p.i., the cells were lysed and subjected to immunoprecipitation by using anti-V5 antibody. Precipitates were examined by using Western blotting with anti-V5 tag antibody (top and third panels) or anti-Flag tag antibody (second and bottom panels). The top two panels show Western blot analysis of the input lysates.

Le May et al. proposed that interaction of NSs with SAP30 interferes with the recruitment of co-activator protein CBP at activation sites on the IFN-β promotor, leading to the inhibition of IFN-β mRNA transcription [11]. To investigate the ability of MP-12-R16H/M250K or MP-12-M250K to suppress IFN-β mRNA transcription, we examined the levels of IFN-β mRNA in MRC-5 cells infected with MP-12, MP-12-ΔNSs, MP-12-M250K or MP-12-R16H/250K at various times p.i. (Fig 5A). MP-12ΔNSs replication induced robust IFN-β mRNA transcription, whereas IFN-β mRNA was not detectable in MP-12- or MP-12-M250K-infected cells. In contrast, low levels of IFN-β mRNA accumulation occurred in MP-12-R16H/M250K-infected cells, demonstrating that the NSs-R16H/M250K was unable to efficiently inhibit IFN-β mRNA transcription. MP-12-M250K replicated as efficiently as did MP-12 in MRC-5 cells, whereas the virus titer of MP-12ΔNSs was significantly lower than that of MP-12 at 72 h p.i. (Fig 5B), demonstrating the efficient inhibition of IFN-β production in the former, but not in the latter. Although MP-12-R16H/M250K replicated better than MP-12ΔNSs, it replicated less efficiently than MP-12, suggesting to us that the low levels of IFN-β production in the MP-12-R16H/M250K-infected MRC-5 cells prevented efficient replication of the virus.

Next, we examined the interactions of the NSs mutants with SAP30. To detect NSs, we used viruses carrying an NSs with a C-terminal Flag tag (MP-12-NSs-Flag, MP-12-M250K-Flag and MP-12-R16H/M250K-Flag). Cells, transiently expressing a V5-tagged SAP30, were infected with these viruses, and the co-localization of the mutated NSs protein with SAP30 was examined by fluorescence microscopy analysis. Like wt NSs, both NSs mutants formed filament-like structures in the nucleus (Fig 5C). The expressed SAP30 was mainly observed in the nucleus and was co-localized with wt NSs and both mutated NSs in the filaments. We also performed co-immunoprecipitation analysis to examine NSs-SAP30 interaction. As a negative control, V5-tagged Venus was expressed in place of V5-tagged SAP30. Anti-V5 antibody co-precipitated wt NSs and both NSs mutants along with V5-tagged SAP30, while the amounts of NSs-M250K that were co-immunoprecipitated with SAP30 were lower than those of wt NSs (Fig 5D). Anti-V5 antibody did not co-precipitate wt NSs along with V5-tagged Venus (Fig 5D). These data demonstrate that both NSs mutants bound to SAP30 in infected cells.

Taken together, these data indicated that NSs-R16H/M250K was unable to completely block IFN-β transcription despite its ability to bind to SAP30.

### Effects of mutations in NSs on its host transcription inhibition activity

**Fig 6:**
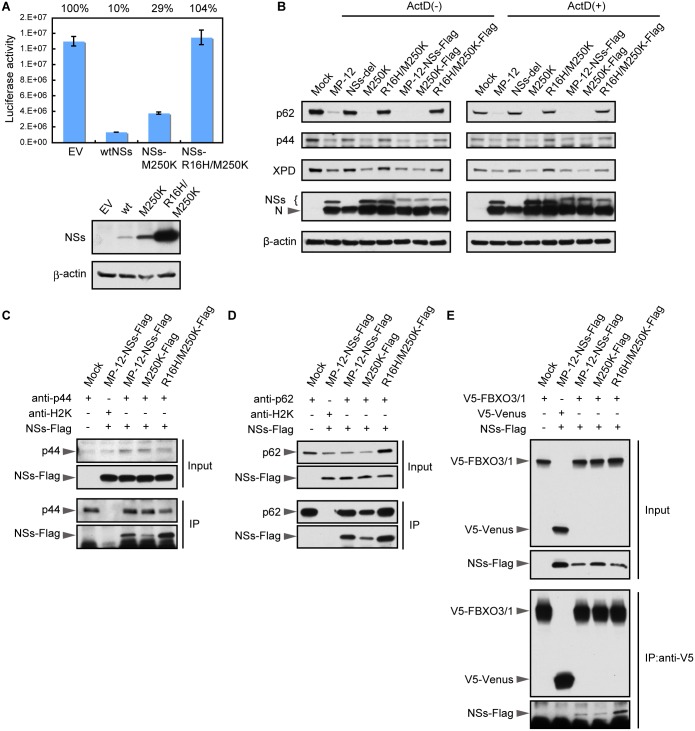
Effect of mutations in NSs on its interaction with TFIIH subunits. (A) HeLa cells were co-transfected with NSs expression plasmid or empty vector (EV) along with reporter plasmid encoding Renilla luciferase. Luciferase activities (relative light units) were measured at 24h post transfection (top panel). Whole cell lysates were prepared from the transfected cells described above and subjected to Western blot analysis by using anti-MP-12 and anti-β-actin antibodies (bottom panel). (B) HeLa cells were mock infected (Mock) or infected with MP-12 or its mutants at an m.o.i. of 3 and harvested at 16 h p.i. Whole-cell lysates were subjected to Western blot analysis by using anti-p62 antibody and p44 antibody, anti-XPD antibody, anti-MP-12 antibody or anti-β-actin antibody as primary antibodies. (C) HeLa cells were mock infected (Mock) or infected with the MP-12 carrying Flag-tagged NSs or NSs or mutant MP-12 carrying Flag-tagged NSs mutant at an m.o.i. of 3. At 8 h p.i., the cells were lysed and subjected to immunoprecipitation by using anti-p44 antibody. Precipitates were subjected to Western blotting and analyzed by using anti-p44 antibody (third panel) and anti-Flag tag antibody (bottom). The top two panels show Western blot analysis of the input lysates by using anti-p44 antibody (top panel) and anti-Flag tag antibody (second panel). (D) HeLa cells were mock infected (Mock) or infected with the MP-12 carrying Flag-tagged NSs or NSs or mutant MP-12 carrying Flag-tagged NSs mutant at an m.o.i. of 3. At 5 h p.i., the cells were lysed and subjected to immunoprecipitation by using anti-p62 antibody. Precipitates were subjected to Western blotting and analyzed by anti-p62 antibody (third panel) and anti-Flag tag antibody (bottom). The top two panels show Western blot analysis of the input lysates using anti-p62 antibody (top panel) and anti-Flag tag antibody (second panel). (E) HeLa cells were transfected with the plasmid encoding V5-tagged full-length FBXO3 or V5-tagged Venus protein and infected with the MP-12 carrying Flag-tagged NSs or mutant MP-12 carrying Flag-tagged NSs mutant at 16 h post transfection. At 8 h p.i., the cells were lysed and subjected to immunoprecipitation using anti-V5 antibody. Precipitates were subjected to Western blotting analysis by using anti-V5 antibody (third panel) and anti-Flag tag antibody (bottom panel). The top two panels show Western blot analysis of the input lysates using anti-V5 antibody (top panel) and anti-Flag tag antibody (second panel).

Both NSs mutants retained some NSs functions, including degradation of PKR and SAP30 binding, and yet these NSs mutants and wt NSs showed different levels of general transcriptional suppression activities in virus-infected cells. Transcriptional suppression activities of the mutated NSs were also examined in cells transiently expressing NSs along with Renilla luciferase from co-transfected plasmids. Luciferase activity was strongly inhibited in the cells co-expressing wt NSs and Renilla luciferase (Fig 6A). Consistent with the data obtained from infected cells (Fig 2), NSs-R16H/M250K expression did not inhibit luciferase activity, whereas NSs-M250K expression moderately inhibited the luciferase activity. Western blot analysis of nuclear and cytoplasmic fractions from virus-infected cells showed that like wt NSs, NSs-R16H/M250 and NSs-M250K were also distributed in both the nucleus and the cytoplasm (S1 Fig), demonstrating that these mutations did not affect the subcellular localization of NSs.

These data prompted us to further examine the interplay between these NSs mutants and a form of host transcription machinery, TFIIH, as other studies have shown an association of TFIIH with NSs in the NSs-induced host transcription shut-off. RVFV NSs binds to p44, a subunit of TFIIH, and interferes with the formation of the TFIIH complex by inhibiting the subsequent interaction of p44 and XPD [12]. The reduction in the abundance of XPD and p44 also occurs upon RVFV infection, although the mechanisms that govern the reduction of these proteins are unclear [12]. In addition, NSs binds to both p62, a TFIIH subunit, and FBXO3, a component of E3 ubiquitin ligase, which leads to p62 degradation [13, 14].

We first examined the abundance of TFIIH components, including p44, p62 and XPD, in infected cells. Substantial reduction of p62 abundance and moderate reductions in the abundances of p44 and XPD occurred in cells infected with MP-12, or MP-12-M250K at 16 h p.i. (Fig 6B, left panels). In contrast, there was no substantial reduction in the abundance of these TFIIH components in cells infected with MP-12ΔNS or MP-12-R16H/M250K. The same results were obtained when viruses carrying Flag-tagged NSs were used. To exclude the possibility that different transcription suppression activities of these viruses affected the results, we repeated the experiments in the presence of ActD and obtained similar results (Fig 6B, right panels).

Next, we tested the interaction of mutated NSs with p44 and p62. Cells infected with MP-12 or its mutants, all of which carried Flag-tagged NSs, were subjected to co-immunoprecipitation analysis using anti-p44 antibody (Fig 6C). Anti-p44 antibody co-immunoprecipitated wt NSs and both mutant NSs along with p44, whereas control anti-H2K antibody precipitated neither p44 nor NSs. These data demonstrated that both NSs mutants bound to p44. To examine the interaction of p62 and NSs, cells were infected with the viruses as described above, and cell extracts were prepared at 5 h p.i., when p62 was still detectable in MP-12-NSs-Flag-infected cells (Fig 6D). Anti-p62 antibody, but not anti-H2K antibody, co-immunoprecipitated wt NSs and both mutant NSs along with p62, demonstrating binding of the mutant NSs proteins to p62. We noted that the amounts of NSs-M250K that were co-immunoprecipitated with p44 and with p62 were lower than those of wt NSs, implying that the binding efficiencies of NSs-M250K for p44 and for p62 were lower than those for wt NSs.

FBXO3 is an interactor of RVFV NSs that is engaged in the degradation of p62; NSs interacts with the full-length FBXO3 protein (FBXO3/1) as well as with a shorter splice variant of the FBXO3 that lacks the C-terminal acidic domain and poly(R) region [14]. Because NSs-R16H/M250K did not induce p62 degradation regardless of its ability to bind to p62 (Fig 6D), we suspected that NSs-R16H/M250K would not interact with FBOX3. To test this possibility, cells transiently expressing the V5-tagged FBXO3/1 were mock infected or infected with MP-12-NSs-Flag, MP-12-M250K-Flag, or MP-12-R16/M250K-Flag. At 5 h p.i., cell extracts were prepared and subjected to co-immunoprecipitation analysis by using anti-V5 antibody (Fig 6E). All of the NSs proteins, including NSs-R16/M250K, were co-immunoprecipitated with FBXO3/1. Table 2 summarizes interactions of the NSs mutants with p44, p62 and FBXO3.

**Table 2:** Summary of the interaction profile of mutated NSs proteins with TFIIH subunits.

	Transcription inhibition	p44	p62		FBXO3
		Binding	Binding	Degradation	Binding
**wtNSs**	**Strong**	**+**	**+**	**+**	**+**
**NSs-M250K**	**Moderate**	**+**	**+**	**+**	**+**
**NSs-R16H/M250K**	**No inhibition**	**+**	**+**	-	**+**

### Virulence of MP-12-R16H/M250K and MP-12-M250K in young mice

**Fig 7:**
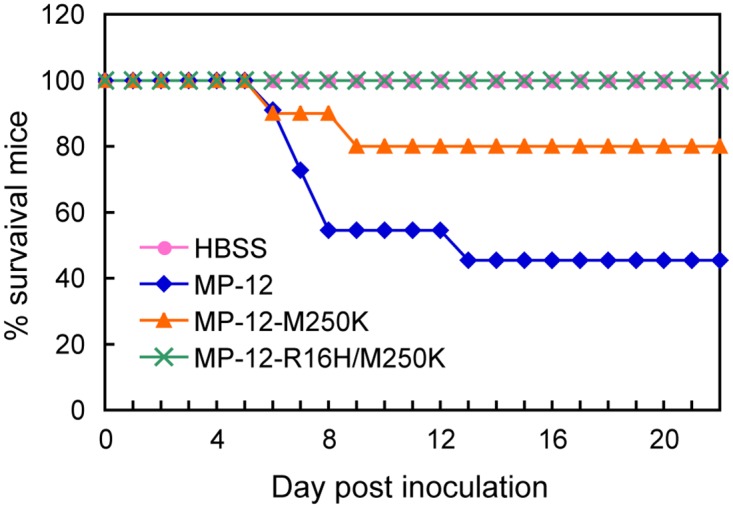
Attenuated virulence of MP-12 NSs mutants. Eighteen-day-old CD1 mice were intraperitoneally inoculated with 10^4^ PFU of MP-12 (n = 11), MP-12-M250K (n = 10) or MP-12-R16H/M250K (n = 10) or inoculated with HBSS (n = 11). The mice were observed for 22 days after inoculation.

NSs is a major virulence factor of RVFV [31]. The NSs-mediated inhibition of type I IFN production is thought to contribute to the virulence of the virus, yet it remains unclear whether the general transcription suppression function of NSs contributes to this virulence. We examined the importance of the NSs-mediated transcription inhibition in RVFV virulence by using a young mouse model. Although MP-12 is known as an attenuated strain, the intraperitoneal inoculation of 10^4^ PFU of MP-12 into 18-day-old CD1 mice resulted in the death of 55% of the mice within 13 days p.i. (Fig 7). Under the same experimental conditions, none of the MP-12-R16H/M250K-inoculated mice and 20% of the MP-12-M250K-inoculated mice died. No obvious clinical signs, including neurological symptoms, were observed. These data demonstrated that the MP-12-R16H/M250K lacked virulence, and MP-12-M250K was less virulent than MP-12 in this young mouse model.

## Discussion

In this study, we demonstrated that the M250K and R16H/M250K mutations in NSs differentially reduced its inhibitory activity on host transcription without affecting its ability to inhibit IFN-β transcription, through its interaction with SAP30, and induced PKR degradation. NSs-R16H/M250K, which completely lacked the activity to inhibit general transcription, correspondingly lost the ability to promote the degradation of p62. Unexpectedly, NSs-R16H/M250K was able to interact with FBOX3 and p62 (Fig 6). These data possibly suggest that the FBXO3-NSs-p62 interaction may not be sufficient to trigger p62 degradation. NSs-M250K exhibited a partially reduced activity to inhibit general transcription. The results of co-immunoprecipitation assays showed that a reduced amount of NSs-M250K co-precipitated with SAP30, p62 and p44, compared with that in wt NSs (Figs 5 and 6). However, the slightly impaired ability of NSs-M250K to inhibit general transcription could not be solely attributed to the lower binding efficiency of NSs-M250K to p44 because NSs-M250K efficiently suppressed IFN-β transcription and induced p62 degradation, despite its lower efficiency of binding to SAP30 and p62. Moreover, NSs-R16H/M250K, which lacked transcription suppression activity, efficiently interacted with p44 (Fig 6). These data bring into question the importance of NSs-p44 interaction for its host transcriptional shut-off function. One possibility is that the NSs-p44 interaction may only make a modest contribution towards its transcription inhibition activity and possibly could have additional, as yet unidentified, biological function(s). A second possibility is that only wt NSs, but not the mutated NSs, is able to interfere with the formation of an active TFIIH complex. NSs competes with XPD for binding to p44, resulting in inhibition of the TFIIH complex formation [12]. Although both NSs-M250K and NSs-R16H/M250K retained the ability to bind to p44, it is possible that the binding of these mutated NSs to p44 did not exclude the binding of XPD.

The R16H single mutation did not affect the ability of NSs to inhibit transcription (Fig 2). Although the M250K mutation is in close proximity to the ΩXaV motif located at the C-terminal region of NSs, which is essential for p62 degradation [32], MP-12-M250K still retained the ability to degrade p62 (Fig 6). However, the R16H/M250K double mutant lost the ability to induce p62 degradation. These results imply that the combined mutations, R16H and M250K, induced an unfavorable structural alteration in NSs, which abolished its function to degrade p62.

MP-12-R16H/M250K replication induced low levels of IFN-β mRNA (Fig 5), indicating that NSs-R16H/M250K was not able to block IFN-β mRNA synthesis as efficiently as wt NSs despite its ability to interact with SAP30 (Fig 5). It has been reported that NSs-induced p62 degradation contributes to the inhibition of IFN-β production [14]. Accordingly, our data suggested that MP-12-R16H/M250K was unable to completely block IFN-β mRNA synthesis due to a lack of ability to promote the degradation of p62 (Fig 6). Although MP-12-R16H/M250K replication induced IFN-β mRNA synthesis, the level of the IFN-β mRNA was significantly lower than that in MP-12ΔNSs-infected cells (Fig 5), suggesting the importance of interaction of NSs with SAP30 for IFN-β inhibition. Taken together, the data shown here and those of others [11, 14] strongly imply that NSs-SAP30 interaction and NSs-induced general host transcriptional suppression function are both necessary for efficient inhibition of IFN-β mRNA transcription.

MP-12ΔNSs or MP-12 encoding a reporter gene in place of the NSs gene causes less prominent cytopathic effects than does MP-12, demonstrating the contribution of the NSs towards the induction of cytotoxicity [24]. MP-12 replication induces NSs-dependent p53 stabilization, which contributes to virus-induced cell death [21], and yet it was unclear which function of the NSs contributed to the induction of the p53-mediated cytotoxicity. As shown in Fig 3, MP-12-infected cells showed the lowest cell viability, followed in order by MP-12-M250K, which moderately suppressed host general transcription, and MP-12-R16H/M250K, which did not suppress host transcription. Hence, there was a correlation between the strength of NSs-mediated, host transcriptional shut-off activity and cell viability in the infected cells (Table 3). Likewise, accumulation of p53 was the highest in MP-12-infected cells, followed in order by that in MP-12-M250K-infected cells and MP-12-R16H/M250K-infected cells, suggesting to us that p53 stabilization also correlates with the transcription inhibition activities of the different NSs mutants. These results indicate that the NSs-mediated host transcriptional shut-off triggered p53 stabilization, leading to p53-mediated cell death.

**Table 3:** Summary of the phenotypic properties of MP-12 carrying mutated NSs.

	PKR degradation	SAP30 Binding	Transcription inhibition	Inhibition of IFN-β mRNA transcription	p53 stabilization	Cytotoxicity (% cell viability)	Virulence in young mice (% survival)
**MP-12**	**Yes**	**Yes**	**Strong**	**Strong**	**Strong**	**10**	**45**
**MP-12-M250K**	**Yes**	**Yes**	**Moderate**	**Strong**	**Moderate**	**61**	**80**
**MP-12-R16H/M250K**	**Yes**	**Yes**	**No inhibition**	**Moderate**	**Weak**	**89**	**100**

Although MP-12 is an attenuated RVFV strain, 55% of 18-day-old CD1 mice died after intraperitoneal inoculation with 10^4^ PFU within 13 days p.i. (Fig 7). As the immune system is not yet fully developed in young mice, it likely failed to prevent systemic infection by MP-12. Consistent with this notion, intraperitoneal inoculation of MP-12 into severe combined immune deficiency mice also caused 100% mortality [33]. MP-12-R16H/M250K carrying NSs that lacks the host transcription inhibition function was completely attenuated in 18-day-old CD1 mice (Fig 7). MP-12-M250K carrying NSs with an impaired ability to inhibit transcription also exhibited reduced virulence when compared to that in its parental virus MP-12. These data highlight the role of the host transcription inhibition function of NSs in RVFV virulence.

Type I IFN has been shown to play a critical role in protecting the host from RVFV-induced disease in animal models [34]. Notably, both the NSs mutants, carrying either the M250K single mutation or the R16H/M250K double mutation, retained the ability to bind to SAP30, the factor that is targeted by NSs to inhibit IFN-β mRNA transcription. However, the induction of IFN-β mRNA synthesis was not completely blocked in MP-12-R16H/M250K-infected MRC-5 cells, most probably due to the lack of inhibition of host transcription in these cells expressing the mutated NSs. These data suggest the possibility that the inability of MP-12-R16H/M250K to completely block the production of IFN-β in infected mice could have contributed towards its attenuation. In addition, the production of other antiviral and/or proinflammatory cytokines could have also contributed towards the attenuated phenotype of both of these mutant viruses, carrying NSs with an impaired ability to block host transcription. It is also possible that the lower levels of virus-induced cytocidal effects might have contributed to the lower virulence of these NSs mutant viruses, as both mutant viruses caused less severe cytopathic effects and cytotoxicity than did MP-12 in cultured cells (Fig 3). We believe that experiments using RVFV mutants, carrying the M250K single mutation and the R16H/M250K double mutation, in animal models would yield valuable information about the role of NSs-mediated host transcription inhibition in regulating host cytokine responses and its impact on the pathogenesis of RVFV. MP-12 is an attenuated live vaccine candidate, but it still harbors residual virulence in a young mouse model. Although further studies are required to test the immunogenicity and protective efficacy of MP-12-M250K and MP-12-R16H/M250K, there is a potential for developing these MP-12-derived NSs mutant viruses as safer live attenuated RVFV vaccine candidates.

We found that the serial passage of an MP-12-derived mutant virus having a large deletion in the 5’ untranslated region of M RNA segment [30] in Vero E6 cells resulted in accumulation of variant viruses that were able to replicate better than the original mutant virus. Most of viruses in passage 18 had a large internal deletion in the NSs gene, which may mean their NSs proteins are biologically inactive. Others have reported that RVFV carrying large deletions in the NSs gene start accumulating from the 15^th^ serial passage in BHK cells of the RVFV P strain, which is defective in IFN-α/β signaling [35]. In our experiment, the large deletions in the NSs gene were detected after the 5^th^ serial passage of the virus. Although the cell lines used in the studies were different, the deletion of the NSs gene as early as after 5 passages implied that there was a selective pressure to remove the NSs gene from the virus genome during the passaging of the mutant virus. The full-length NSs gene of the uncloned passage 18 viruses had M250K, M250T, K202N and D100G mutations (Table 1). In the cells infected with MP-12 carrying NSs with a D100G mutation, the accumulation of NSs was poor (Fig 1), indicating that the D100G mutation affects the efficient accumulation of NSs. As two out of the five plaque-cloned viruses (clones 3 and 4, Table 1) also had the D100G mutation in NSs, this possibly affected its accumulation in infected cells. The remaining three clones carried NSs with a M250K or R16H/M250K mutation. Our results showed that these mutations in NSs partially or completely abolished its host transcription inhibition function without affecting its ability to interact with SAP30 to inhibit IFN-β mRNA synthesis. These data implied that the host transcription function of NSs was unfavorable for the mutant virus, carrying a deletion in the 5’ UTR, to replicate well in VeroE6 cells. MP-12-M250K and MP-12-R16H/M250K replication induced low cytotoxicity due to its lower inhibitory activity on host transcription (Figs 2 and 3). We suspect that the lack of NSs-induced host transcription suppression created a favorable cellular environment for the mutant virus, thereby allowing the emergence and accumulation of mutant viruses that lacked this function. Further studies are required to delineate the importance of NSs-mediated host transcription inhibition in the virus life cycle.

## Supporting Information

S1 Fig Subcellular localization of NSs mutants in infected cells. HeLa cells were infected with MP-12-NSs-Flag or its mutants, at an m.o.i. of 2 and at 6 h p.i., the cells were lysed using the lysis buffer (10 mM Tris-HCl, pH 7.5, 10 mM KCl, 1.5 mM MgCl_2_, 0.5% Triton X-100, protease inhibitor cocktail) followed by incubation on ice for 10 min. After centrifugation at 2,000 x g for 2 min, the resulting supernatant was collected as the cytoplasmic fraction (C). The pellet from this centrifugation was washed once with the lysis buffer (without Triton X-100), suspended in 1X SDS sample buffer and denoted as the nuclear fraction (N). The subcellular fractions were analyzed by Western blotting using anti-Flag, anti-HSP90 and anti-Lamin A antibodies.
(TIF)
