# Supplementary figures and images for: Mechanistic Insight into the Host Transcription Inhibition Function of Rift Valley Fever Virus NSs and Its Importance in Virulence
Source: PLoS Negl Trop Dis. 2016 Oct 6;10(10):e0005047. doi: 10.1371/journal.pntd.0005047 (PMC5053439; doi:10.1371/journal.pntd.0005047)

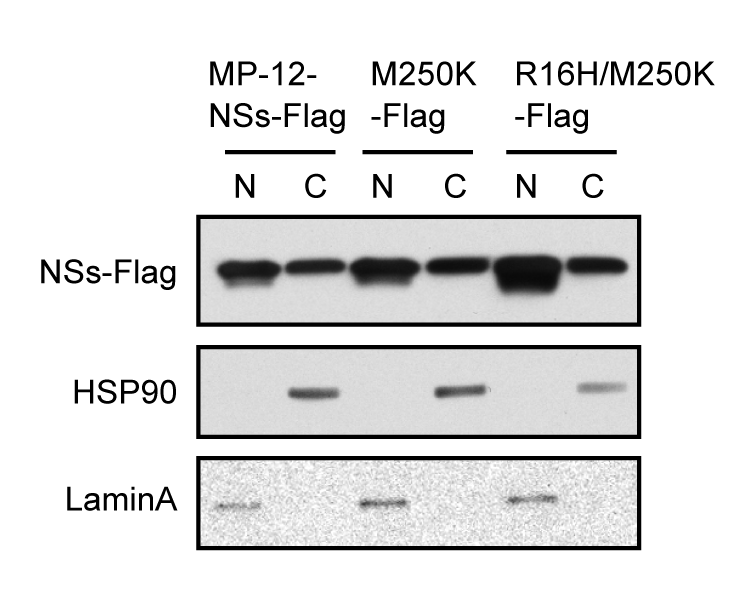

Supplement: S1 Fig — HeLa cells were infected with MP-12-NSs-Flag or its mutants, at an m.o.i. of 2 and at 6 h p.i., the cells were lysed using the lysis buffer (10 mM Tris-HCl, pH 7.5, 10 mM KCl, 1.5 mM MgCl2, 0.5% Triton X-100, protease inhibitor cocktail) followed by incubation on ice for 10 min. After centrifugation at 2,000 x g for 2 min, the resulting supernatant was collected as the cytoplasmic fraction (C). The pellet from this centrifugation was washed once with the lysis buffer (without Triton X-100), suspended in 1X SDS sample buffer and denoted as the nuclear fraction (N). The subcellular fractions were analyzed by Western blotting using anti-Flag, anti-HSP90 and anti-Lamin A antibodies. (TIF) [file pntd.0005047.s001.tif]
